# Systemic Therapy for Gastrointestinal Stromal Tumor: Current Standards and Emerging Challenges

**DOI:** 10.1007/s11864-022-00996-8

**Published:** 2022-08-17

**Authors:** Wen-Kuan Huang, Chiao-En Wu, Shang-Yu Wang, Ching-Fu Chang, Wen-Chi Chou, Jen-Shi Chen, Chun-Nan Yeh

**Affiliations:** 1 Division of Hematology-Oncology, Department of Internal Medicine, Chang Gung Memorial Hospital at Linkou, Taoyuan, Taiwan; 2 College of Medicine, Chang Gung University, Taoyuan, Taiwan; 3 Department of Surgery and GIST team, Chang Gung Memorial Hospital at Linkou, Taoyuan, Taiwan

**Keywords:** Gastrointestinal stromal tumors, Imatinib, Sunitinib, Regorafenib, Ripretinib, Avapritinib, Systemic treatment

## Abstract

Gastrointestinal stromal tumor (GIST), though rare, is the most common mesenchymal tumors of the gastrointestinal tract. *KIT* or *PDGFRα* mutation plays as an oncogenic driver in the majority of GISTs. Surgical resection is the only curative treatment for localized disease. The discovery of imatinib with promising anti-tumor effect and successive tyrosine kinase inhibitors (TKI), including second-line sunitinib and third-line regorafenib, revolutionized the management of advanced and metastatic GIST over the past two decades. Recently, ripretinib and avapritinib were approved for the fourth line setting and for PDGFRA exon 18-mutant GIST in first-line setting, respectively. Despite multi-line TKIs exerted ability of disease control, drug resistance remained an obstacle for preventing rapid disease progression. Experimental TKIs or novel therapeutic targets may further improve treatment efficacy. Immune checkpoint inhibitors such as anti-programmed cell death protein-1 (PD1) and anti-CTL-associated antigen 4 (CTLA-4) showed moderate response in early phase trials composed of heavily pretreated patients. *KIT/PDGFRα* wild-type GISTs are generally less sensitive to imatinib and late-line TKIs. Recent studies demonstrated that targeting fibroblast growth factor receptor signaling may be a potential target for the wild-type GISTs.

## Introduction

Gastrointestinal stromal tumors (GISTs) are a subgroup of mesenchymal tumors arising from any location of the gastrointestinal tract. The most common sites of GISTs are stomach followed by small intestine. GISTs were ever considered as smooth muscle tumors (such as leiomyomas or leiomyosarcomas) based on the histologic characteristics [1]. Until the discovery of KIT(CD117) expression in GIST[2], the origin of GIST was proposed to be from the interstitial cells of Cajal (ICCs) which are the pacemaker cells of gastrointestinal tract [3–5]. The nature of GISTs became better understood with the identification of KIT(CD117) expression and c-KIT mutations [2, 6]. Thereafter, c-KIT mutations [2, 6] followed by platelet-derived growth factor receptor alpha (PDGFRA) mutations [7] were discovered in GISTs, so small molecule targeted therapies with tyrosine kinase inhibitors [8, 9] were developed in GIST. Imatinib, sunitinib, regorafenib, and ripretinib are approved in advanced GIST and imatinib is approved for high-risk GIST as adjuvant treatment.

Although most GISTs have either mutation of KIT or PDGFRA kinase genes as driver mutations, approximately 10 % of GISTs do not harbor a KIT or PDGFRA mutation, which are collectively grouped as KIT/PDGFRA-wild type (WT) GIST. Patients with KIT/PDGFRA-WT GIST may have primary resistance to imatinib. A PDGFRA exon 18 D842V mutation is another primary resistance mechanism. For patients with symptomatic and/or rapidly progressive disease harboring a PDGFRA exon 18 D842V mutation, avapritinib has been suggested over either imatinib or observation in the setting of initial therapy [10••].

In this review, we comprehensively reviewed the current development of medical treatment in terms of distinct treatment settings (palliative treatment for advanced/metastatic GIST, adjuvant treatment for high-risk GIST, and neoadjuvant treatment for locally advanced GIST) and molecular profiling (KIT, PDGRFA, and WT). We further explored the experimental tyrosine kinase inhibitors and emerging drug development.

## Neoadjuvant settings

The primary goal of preoperative imatinib is to reduce tumor size, maximize resectability, and improve quality of life due to avoidance of multi-visceral resection.

Several prospective trials reported the efficacy of imatinib use before surgery, however, the results of survival analysis should be interpreted with caution because of the subsequent use of adjuvant imatinib in these studies [11–13]. The first prospective phase II trial (RTOG 0132/ACRIN 6665) observed that 7% and 83% of patients with potentially resectable GIST (*n*=30) receiving 600mg imatinib daily reached partial response and stable disease, respectively [11]. In one study from the MD Anderson Cancer Center, 19 patients were randomized to receive imatinib (600 mg daily) within 3-7 days. This study showed rapid tumor response with the response rate of 71% by CT scan [12]. In the subgroup analysis of the phase III BFR14 trial, neoadjuvant imatinib yielded a response rate of 60% (15 of 25 patients) after a median of 7.3 months of imatinib treatment [13]. An Asian multi-center phase II trial revealed that 6-9 months of preoperative imatinib for patients with gastric GIST ≥10cm could reach R0 resection of 91% [14]. The phase II APOLLON study recruited patients with locally advanced but potentially resectable GISTs (*n*=41) [15]. Imatinib 400 mg daily for 6 months was administered before surgical resection. Thirty-four out of 41 patients (83%) underwent primary tumor resection, with 88% of patients (30/34) achieving R0 resection. Of note, the median 3-year progression-free survival (PFS) was 85.2% with no adjuvant imatinib use in all patients.

Given many patients who were considered neoadjuvant imatinib were with intermediate- or high-risk GIST, adjuvant imatinib for 3 years may also contribute to overall survival (OS) benefit. One real-world data utilizing National Cancer Database showed no OS differences when comparing neoadjuvant imatinib followed by surgery and adjuvant imatinib with patients received upfront surgery followed by adjuvant imatinib [16]. While several single-arm prospective studies have demonstrated clinical benefit of neoadjuvant imatinib, to date, no randomized phase III data was available to assess the efficacy and safety of neoadjuvant imatinib in locally advanced GIST. The optimal duration of neoadjuvant imatinib is also not confirmed. However, our prospective study suggested that a median duration of 6.1 months imatinib use can achieve the maximal shrinkage of tumor [17]. In conclusion, we recommended to assess the necessity of neoadjuvant imatinib for locally advanced or potentially resectable GIST on an individual basis. Careful monitoring of complications of neoadjuvant imatinib use is necessary.

## Adjuvant settings

Although surgical resection is the curative treatment for localized GIST, surgery alone can not prevent relapse in patients with high-risk features. Before the era of adjuvant imatinib for high-risk GIST, about 50% patients after primary resection developed recurrence with the median time to recurrence of 2 years [18, 19]. The role of adjuvant imatinib (400mg daily) for 1 year was confirmed in the phase III trial (ACOSOG Z9001) [20]. Risk of recurrence was substantially reduced by 40% (hazard ratio [HR], 0.6; 95% CI, 0.43 to 0.75; adjusted *p*< 0.001) in imatinib arm. There was no overall survival (OS) difference between imatinib and placebo arms. Further analyses showed that KIT exon 11 deletions of any type but not exon 11 insertion or point mutation had better recurrence-free survival (RFS). KIT exon 9, PDGFRA mutation, or wild-type GIST were not associated with better RFS in the imatinib treatment arm.

The optimal duration of postoperative imatinib was investigated in following trials. EORTC-62024 trial compared 2-year imatinib with observation in 835 patients with localized intermediate- or high-risk GIST [21]. The 5-year imatinib failure-free survival did not show significant difference (87% in the imatinib arm versus 84% in the control arm, HR, 0.79; 98.5% CI, 0.50 to 1.25; *p*= .21). Nevertheless, imatinib treatment was associated with favorable 3-year RFS (84% vs. 66%, log-rank *p*< .001) and 5-year RFS (69% vs. 63%, log-rank *p*<.001). Furthermore, the Scandinavian Sarcoma Group XVIII/AIO trial demonstrated the survival benefits of 3 years of imatinib (400mg daily) compared with 1-year imatinib use in high-risk patients. The 5-year RFS was 71.1% versus 52.3% (HR, 0.60; 95% CI 0.44 to 0.81; *p*< .001) [22]. Long-term OS benefit remained for 3-year imatinib use (10-year OS: 81.6% vs. 66.8%, HR: 0.5, 5% CI, 0.32–0.80; *p*<0.003) [23••].

Further analysis of mutation pattern on RFS showed that KIT exon 11 deletions on codons 557 and/or 558 had most of the benefit from 3-year adjuvant imatinib. This benefit was not found in exon 9 mutations, PDGFRA mutation, or wild-type GISTs [24], which is in line with the mutation analysis of ACOSOG Z9001. These results lead to a subsequent question whether 5-year imatinib use after resection would be better in terms of RFS and OS. The phase II trial (PERSIST-5) reported the 5-year DFS of 90% (95% CI, 80%-95%) in patients with resected GIST who received 5 years of imatinib therapy [25]. A phase III clinical trial (SSGXXII/AIO, NCT 02413736) is aiming to investigate the survival difference between 5- and 3-year of adjuvant imatinib. Another phase III trial (ImadGIST, NCT02260505) is recruiting to address the impact of 6-year imatinib compared with standard 3-year imatinib.

## Systemic treatments for advanced/metastatic GIST

**Fig. 1:**
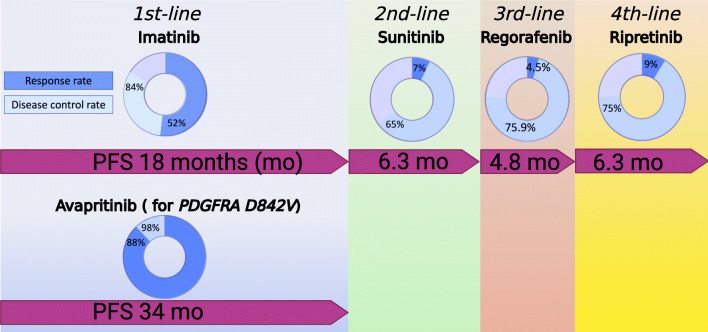
Treatment scheme of FDA-approved tyrosine kinase inhibitors for advanced GISTs. Figure created with BioRender.com.

While targeted therapy is the mainstay of systemic treatments for GIST, the treatment paradigm for advanced/metastatic GIST continues to evolve due to recent approval of two new tyrosine kinase inhibitors (TKIs). To date, imatinib as the first-line treatment is irrefutable based on its durable efficacy and favorable safety. Of note, a subset of patients harboring mutations which are known to resist to imatinib (PDGFRA exon 18 D842V) have better choice of first-line treatment. Avapritinib is approved in January 2020 as first-line therapy in patients with advanced/metastatic GISTs harboring PDGFRA exon 18 mutations (including D842V). Patients experienced progressive disease on imatinib are suggested to receive second-line sunitinib with a median PFS of about 6–9 months. Regorafenib is the currently established third-line therapy as the longer median PFS of approximately 4–5 months than the placebo arm. Ripretinib, a switch-control TKI by dual binding to both the switch pocket and the activation loop, locks the TKIs in an inactivated state. The phase III trial (INVICTUS) confirmed the role of ripretinib as the fourth-line treatment based on a median PFS of over 6 months compared to only 1 month in the placebo arm. The preliminary results of the phase III INTRIGUE trial showed that ripretinib is not superior to sunitinib as a second-line TKI for patients with GIST in terms of progression-free survival [26]. The relative safety profile similar to imatinib provides heavily pretreated patients a feasible drug of choice. We summarized the current standard TKIs for advanced GIST in Figure 1. For patient progress beyond the approved therapies, several targeted drugs may be considered in certain circumstances. Participating in clinical trials for these patients are encouraged.

### Early approved TKIs: imatinib, sunitinib, and regorafenib (Figure 1)

The amazing case report published in 2000 [27] rapidly led to conduct phase 1 and phase 2 trials to investigate the role of imatinib in advanced GIST [9, 27]. The response rate was 53.7% treated with 400 or 600 mg once daily with a median OS of 57 months regardless of 400 or 600mg starting dose [28]. Eventually, the confirmatory phase 3 trials studied the efficacy and safety of 400 versus 800mg imatinib daily [8, 29]. The comparable efficacy in terms of response and 10-year OS between the two doses leads to the use of 400 mg daily as the standard first-line therapy [30]. A large meta-analysis (MetaGIST) demonstrated a longer PFS from 800 mg versus 400 mg daily for patients with GIST harboring KIT exon 9 mutations (HR=0.89; 95% CI, 0.79 to 1.00) [31]. Approval of sunitinib as second-line setting was granted from a phase III clinical trial with a median PFS of 27.3 weeks versus 6.4 weeks in the placebo arm [32]. The overall response rates were 7% and 0% for the sunitinib and placebo groups, respectively. A total of 50mg once daily with 4-week on and 2-week off is the standard dose. However, continuous daily dose of 37.5mg is also an alternative dosing schedule with less toxicity [33]. In line with the clinical trials, the real-world data demonstrated comparable efficacy and safety of sunitinib in patients resistant or intolerant to imatinib [33]. Regorafenib was approved as third-line treatment because one phase III study showed a median PFS of 4.8 months versus 0.9 months for the placebo group [34]. Of note, the overall response rate was 4.5% and 1.5% in the regorafenib group and the placebo group, respectively. Several retrospective analyses of regorafenib showed a mPFS of 7.7–8.7 months [35–37].

### Impact of mutation status on response

Mutation analysis is critical when evaluating the efficacy of approved TKI in patients with advanced GIST. Patients with primary exon 11 mutation appear to be sensitive to standard dose imatinib (400mg daily), which is less effective for those with exon 9 mutations. Exon 9 mutations may benefit with the escalation of imatinib doses (800mg daily), whereas wild-type GISTs demonstrate a limited response to imatinib. The imatinib response in PDGFRA-mutant GISTs was mostly obtained from subgroup analysis with a small number of patients. Of note, PDGFRA exon 18 D842V is substantially resistant to imatinib from preclinical and clinical studies [38]. This type of mutation is important when imatinib use is administered for neoadjuvant settings. After progression on imatinib treatment, secondary mutations commonly occurred in KIT exon 13, 14, or 17 [32]. Secondary mutations in the activation loop of KIT gene were found in some patients after sunitinib failure [39]. Sunitinib is more effective in KIT exon 13 and 14 mutation (ATP-binding pocket) but not exon 17-18 (activation loop) [40]. In contrast, regorafenib shows higher efficacy for suppressing KIT exon 17 mutations, but is less potent for the exon 13 mutations [41, 42].

The recent approved TKIs, avapritinib, is undoubtedly showing the importance of molecular profiling. Patients with advanced GIST harboring PDGFRA exon 18 mutations, including D842V mutation, are indicated for the avapritinib use. Repritinib demonstrated potent activity towards several primary and secondary KIT mutations across the ATP-binding site (exon 13 and 14) and the activation loop (exon 17 and 18). However, one human GIST T1 cell line with knocked-in PDGFRA D842V was highly resistant to ripretinib [43]. KIT D816V is the homologous mutation corresponding to PDGFRA D842V, suggesting that this mutation is potentially resistant to imatinib. The dynamic evolution after ripretinib failure would need to be elucidated in the near future.

### Wild-type GIST

**Fig. 2:**
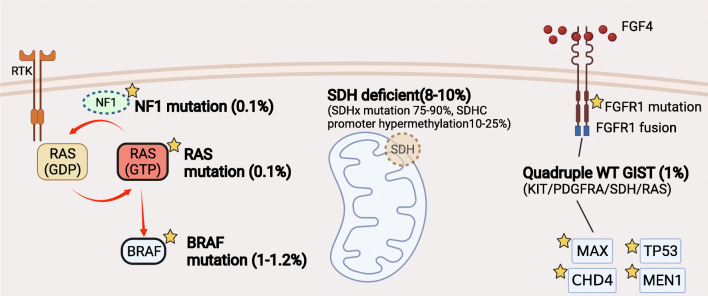
The molecular characterization of KIT/PDGFRA wild-type (WT) gastrointestinal stromal tumor. Dashed outline indicates loss of function, and bold outline indicates activation of kinases Star symbols indicate gene mutations. SDH, succinate dehydrogenase; NF1, neurofibromin 1; RTK, receptor of tyrosine kinase; FGF, fibroblast growth factor; FGFR, fibroblast growth factor receptor; MAX, MYC Associated Factor X; MEN1, Menin 1; CHD4, Chromodomain Helicase DNA Binding Protein 4. Figure created with BioRender.com.

Figure 2 shows the molecular classification of KIT/PDGFRA wild-type gastrointestinal stromal tumor.

There are 10%-15% of GISTs lacking KIT and PDGFRA mutations, called wild-type GISTs (WT GISTs), which is known to be relatively resistant to imatinib. According to the loss of SDHB expression in immunohistochemistry staining, WT GISTs can be further divided into the SDH-deficient and non-SDH-deficient GISTs. In the group of non-SDH-deficient GISTs, some are associated with RAS-MAPK pathway alterations such as gain-of-function RAS/BRAF mutations or loss of neurofibromatosis type 1 (NF1) mutations. The rest of non-SDH-deficient GISTs was classified as quadruple wild-type GIST (KIT/PDGFRA/SDH/RAS pathway wild-type). SDH-deficient GIST is the majority type of WT GISTs. In a large WT GIST cohort (*n*=95), 88% (*n*=84) were SDH-deficient GIST including 63 patients with SDHX gene mutations and 25 patients with SDHC promoter methylation [44]. Response to imatinib in SDH-deficient GISTs is low, ranging from 2 to 8% [44, 45]. Sunitinib and regorafenib showed higher response with 18.4% and 33.3%, respectively [44, 46]. Understanding the tumorigenesis of SDH-deficient GISTs will help continuing to explore the potential therapeutic targets. Ripretinib was granted for later-line treatment of all molecular types of GIST, including WT GIST. Of note, only seven patients with WT GIST were enrolled in the randomized trial of ripretinib. The effect of ripretinib in WT GIST remains to be determined in studies with a bigger number. Quadruple WT GISTs present marked molecular heterogeneity characterized with overexpression of CALCRL and COL22A1. Other gene alterations in quadruple WT GISTs included fusion genes (ETV6-NTRK3, KIT-PDGFRA, or FGFR1-HOOK3) and mutations (TP53, CDK6, CHD4, and FGFR1) [47]. Of note, the presence of GIST with NTRK fusions has been questioned [48, 49]. Two cases of spindle-cell sarcoma with NTRK fusion have been found with limited morphologic and immunophenotypic features to support the diagnosis of GIST [49, 50]. Recently, FGF4 overexpression was found in quadruple WT GISTs [51•]. FGF4-FGFR1 signaling pathway is predominant in quadruple WT GISTs, which may represent a novel therapeutic target.

### Emerging agents

While there are three-line targeted therapies for standard management of patients with advanced GISTs, resistance to approved agents is common that leads to development a variety of treatment strategies such as targeting switch control of KIT, conformation-specific active state (PDGFRA D842V or KIT), alternative kinases (ex, MET and AXL), and immune microenvironment. Here we will briefly review the updated advances of selected emerging agents.

#### Avapritinib

Avapritinib (BLU-285) is a potent selective type I inhibitor of KIT and PDGFRA, binding to the kinases in their active conformation, which is in contrast to other type II inhibitors including imatinib, sunitinib, regorafenib, and ripretinib. Preclinical studies demonstrated its superior inhibition of PDGFRA 842V and KIT exon 11 combined with 17 mutations [52, 53]. Accordingly, A phase I trial (NAVIGATOR) opened in 2015 to test the anti-tumor activity in advanced GIST with PDGFRA 842V or treated with at least two lines of TKI. The dose escalation part determined the maximum tolerated dose of 400 mg daily while 300 mg daily as the recommended phase II dose (RP2D) due to the toxicity. The initial report of 25 patients with PDGFRA D842V GIST showed tumor regression across all dose levels (30–400 mg orally daily) in 2017. Updated results in 2018 showed that the 56 patients with PDGDRA D842V continued to demonstrate the substantial efficacy with 9% (5/56) complete response (CR) and 79% partial response (PR) (44/56) [10••]. The clinical benefit rate is 98%. Long-term follow-up data revealed the median PFS was 34.0 months (95% CI: 22.9- not reached [NR]) [54•]. The median duration of response was 27.6 months (95% confidence interval [CI]: 17.6-NR). Median OS was not reached. Based on these promising results, avapritinib was approved as the first-line use for patients with advanced GIST harboring PDGFRA D842V.

The role of avapritinib for heavily pretreated advanced GIST was first reported from the NAVIGATOR trial. Overall response rate of 22% was observed in 111 patients (primarily KIT, median 4 prior TKI) treated with avapritinib. The median duration of response was 10.2 months. The superior efficacy of avapritinib as late-line treatment compared with sunitinib and regorafenib leads to the phase III confirmatory trial (VOYAGER). In this open-label, randomized trial of avapritinib (300mg daily) vs regorafenib (160 mg once daily, 3 weeks on and 1 week off) as third-line in patients with advanced GIST, the primary end point was not met ( PFS 4.2 vs. 5.6 months, *p*=0.055) [55•]. The response rate was 17.1 vs 7.2% (*p*=0.03), with durations of responses of 7.6 and 9.4 months for avapritinib and regorafenib, respectively. Of note, this trial showed highly similar efficacy with the GRID study (PFS of 4.8 months and response rate of 4.5%) [34]. The reason to explain “significant tumor response but insignificant PFS benefit” remains to be determined. Several hypotheses have to be considered. First, avapritinib is a relatively selective TKI to specific PDGFRA and KIT mutations given the nature of type I inhibitor (binding the active state of conformation). It might be better to recruit target population with sensitive mutations (enrichment) rather than unselected patients (all-comers). Second, regorafenib is a multi-targeted TKI inhibiting several alternative kinases including VEGFR, which is absent for avapritinib. The anti-angiogenesis effect might slow tumor progression after the presence of regorafenib resistance, leading to the slightly longer PFS. In contrast, rapid progression once resistant to avapritinib might partly explain the slightly shorter PFS. Third, reduced dose of avapritinib due to clinically insignificant laboratory changes, such as elevated total bilirubin or creatine kinase level, during this trial might be relative strict to influence drug efficacy. We need further studies exploring mutation profiling, drug compliance, and dose intensity data from this trial to test our hypothesis. In summary, despite avapritinib failed to demonstrate superior efficacy at the third-line setting compare with regorafenib, the specific potency of avapritinib to inhibit PDGFRA 842V mutation, which is insensitive to imatinib, leads to it recognized as the first TKI ever for this type of mutation.

The mechanism of avapritinib resistance has been explored in four patients with primary PDGFRA D842V mutation who progressed on avapritinib [43]. The secondary mutations occurred at V658A, N659K, Y676C, and G680R (exon 13–15) of PDGFRA in more than one patients refractory to avapritinib. These findings suggest that avapritinib resistance mainly remain depending on the PDGFRA oncogenic signalling.

The safety profile demonstrated that most adverse events (AEs) related to avapritinib were grades 1–2. The common AEs included nausea, fatique, edema, and diarrhea, which were similar to imatinib, sunitinib, or sorafenib. Of note, any-grade cognitive effects occurred in 46% patients (*n*=115) and 26% (*n*=62) in the NAVIGATOR trial and VOYAGER trial, respectively [54•, 55•]. Another major avapritinib-associated AE was intracranial haemorrhage (1.3–2.4%). Overall, the AEs of avapritinib are manageable, while awareness and early management of neurological AEs are important. As the exploratory analysis of the NAVIGATOR trial showed incrementally higher avapritinib dose associated with longer PFS throughout the treatment period [54•], how to balance between the highest tolerable avapritinib dose and reduced dose for toxicity management is essential to maximize the efficacy of avapritinib.

#### Ripretinib

Ripretinib is a broad-spectrum kinase inhibitor with dual blockade of switch pockets (switch control), which is unique to prior TKIs used in GIST. The inhibition of both switches: one inhibitory pocket at the juxtamembrane domain, and the activating switch at the activation loop, prevents the active conformation and stabilizes switch elements in the inactive state. Preclinical studies showed promising inhibition of ripretinib on primary KIT and PDGFRA mutations or combined with secondary mutations across a variety of KIT exon 13,14,17, and 18 [56••]. As heterogeneity KIT mutations and stabilization at active state are two important features in GIST progression upon multi-line TKI use, ripretinib is considered a logical consequence in the late-line settings. Therefore, the encouraging preclinical results bring it into the clinical phase I study in 2015.

In the early dose-escalation part (*n*=68), 20–200mg twice a day or 100–250mg once daily was tested but no maximum tolerated dose was determined. The RP2D of ripretinib was determined as 150mg once daily. In this phase I trial, the 142 patients with advanced GIST received ripretinib at second-line (*n*=31), third-line (*n*=28), and fourth-line (*n*=83). Ripretinib showed more anti-tumor activity in earlier lines. The overall response rate (ORR) and median PFS were 19.4% and 10.7 months at second line, 14.3% and 8.3 months at third line, and 7.2% and 5.5 months at fourth line, respectively. A total of 150mg once daily of ripretinib use was well-tolerated and toxicities were generally manageable with only 5.6% patients discontinued ripretinib due to drug-related adverse events.

The INVICTUS study explored the efficacy of ripretinib in advanced GIST patients who were intolerant or progressed after all three TKIs approved for the treatment of GIST. This phase III, double-blind, trial enrolled129 metastatic GIST patients to either ripretinib (*n*=85) or placebo (*n*=44) [57••]. The median PFS as the primary end point was 6.3 months vervus 1.0 month for ripretinib and placebo, respectively. The ORR of 9% is in line with the results from the phase I trial. While most anti-tumor activity of ripretinib is stable disease (66%), the 6-month and 12-month PFS were 51% and 22.2%. The lengthy anti-tumor effect suggests the potency of ripretinib overcoming heterogeneously second mutations of KIT/PDGFRA or activation of alternative kinases in late-line settings. The safety profile of this phase III trial was in line with the previous phase I trial with reduced dose and dose discontinuation of 6% and 5% of patients, respectively. Overall side effects were low grade and similar to the reports of prior TKIs such as hand foot skin reaction (21%), nausea (26%), fatigue (26%), and diarrhea (20%). Of note, alopecia (49%) is a special adverse event of ripreitnib use. Based on these data, ripretinib was approved by FDA in 2020 for advanced GIST progressed on previous three or more kinase inhibitors.

We continue exploring the role of ripretinib in the management of patients with GIST. The INTRIGUE trial comparing ripretinib with the standard second-line sunitinib in GIST previously treated with imatinib. To date, the preliminary results showed the median PFS was 8.0 months versus 8.3 months (HR, 1.05; nominal *p* =0.715), not reaching the primary end point.

While we still wait for the final analysis of this study, future analysis of mutation profiling in the INVICTUS and INTRIGUE trials may shed light on more understanding of the target populations for ripretnib use.

#### Other experimental agents

**Fig. 3:**
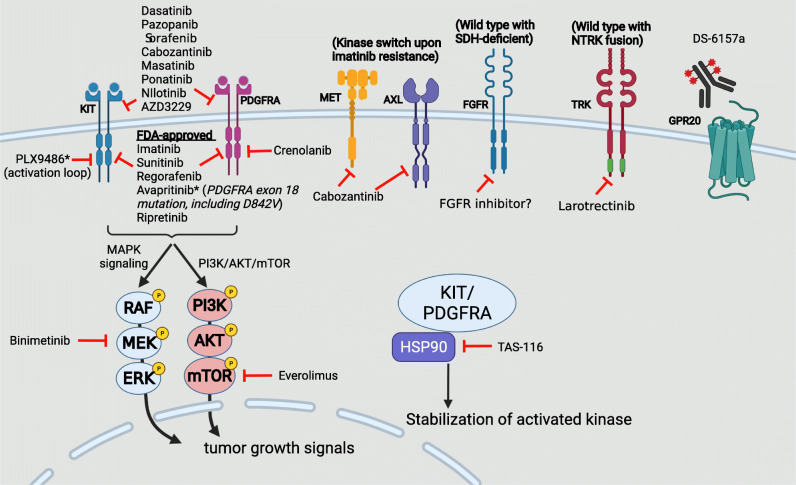
Mechanisms of actions of approved TKI and selected investigational systemic treatments targeting GIST. *Type I TKI (targeting active formation). Figure created with BioRender.com.

Figure 3 shows the mechanisms of action for some novel experimental targeted drugs.

There are many experimental drugs developing for anti-tumor activity of GIST. We only selected some representative drugs for a brief summary. Dasatinib has shown active anti-tumor activity for advanced GIST in single-arm phase 2 trials [58, 59]. However, the less unfavorable safety profile of dasatinib compared with imatinib and seemingly less potent than regorafenib, limiting its further development in GIST. Nilotinib has been shown encouraging results for advanced GIST after failure of both imatinib and sunitinib [60]. Therefore, nilotinib was tested as third-line therapy in a phase III trial. However, the PFS showed no significantly different compared to the control group (median PFS of 3.7 months in both groups) [61]. Sorafenib has shown moderate tumor control activity at the third-line settings [62, 63]. The established role of regorafenib, a similar drug from the same manufacturer, possibly explaining the absence of phase 3 trial of sorafenib. Cabozantinib is active against not only KIT but also VEGFR2, MET, and AXL, which have been shown to play a role of imatinib resistance (kinase switch) [32, 64]. In the EORTC 1317 phase II trial (CaboGIST) study, patients progressive on imatinib and sunitinib were enrolled. The progression-free rate at 12 week was 60%. The median PFS was 6 months [65]. Several other TKIs such as pazopanib, anlotinib and masitinib have been proposed under clinical trial investigation. With the approval of ripretinib for late-line use, the continuation of the development of the aforementioned TKIs is uncertain. AZD3229 is a new selectively KIT/PDGFRA inhibitor, specifically targeting a spectrum of primary and drug-resistant KIT/ PDGFRA mutations observed in GIST [66•]. The superior inhibitory efficacy on primary and activation-loop mutations has been demonstrated in in-vitro and the PDX model [66•, 67], which needs further clinical trial to confirm its role in the treatment of advanced GIST. Recently, PLX9486, a type I inhibitor selectively blocking the active conformation of KIT, presented its efficacy combined with type II inhibitor such as sunitinib or pexidartinib in a phase Ib/IIa trial [68]. With co-targeting both active and inactive conformation of the same kinase (KIT), PLX9486 was associated with a promising survival benefit (mPFS of 12.1 months) and an acceptable safety profile, further confirmatory trials are required to justify these findings.

Therapeutics targeting alternative signaling pathways other than KIT/PDGFRA have been investigated [69]. Heat-shock protein 90 (HSP90) is a chaperone required to maintain proteins stabilization and correct folding, which is critical for activation of KIT and PDGFRA [70]. A HSP90 inhibitor (TAS-116) has demonstrated active activity as fourth-line therapy with a median PFS of 4.4 month [71]. DS-6157a, a first-in-class antibody-drug conjugate assembled with an anti- G protein-coupled receptor 20 (GPR20) antibody, a novel tetrapeptide-based linker, and DNA topoisomerase I inhibitor exatecan derivative (payload). As GPR20 is selectively expressed on GIST cells, DS-6157a showed encouraging preclinical efficacy in GIST xenograft models resistant to imatinib, sunitinib, and regorafenib [72]. Overall, these agents are potential for anti-tumor efficacy based on the preclinical or early phase studies.

### Immunotherapy

Characterization of immune microenvironment of GIST revealed important immunological phenomenon. Blakely et al. showed the expression of PD-L1 was associated with larger size and higher mitotic rate. Higher CD3+CD8+ tumor infiltrating lymphocytes (TILs) was associated with smaller tumor size in PD-L1+/IDO+ GISTs [73]. T cells and macrophages were the two most abundant immune cell types in the microenvironment of GIST [74•, 75]. Pantaleo et al. reported that most GIST samples expressed rich immune infiltration with the T-cell inflamed signature and IFN-γ signature, which were positively correlated with PD-L1 expression [76].

Correlative studies found that imatinib treatment modifies immune microenvironment to enhance immune-boosting activity such as increasing CD8+/Treg ratio, IFN-γ producing CD8+ cells, and suppressing IDO expression [76, 77]. Moreover, mutational influence on tumor microenvironment was observed [78•]. PDGFRA-mutant GISTs possess higher immune signature and immune cell pathway enrichment by gene-expression data compared to other types such as KIT-mutant, SDH-deficient, and NF-1-mutant. Of note, PDGFRA- and KIT-mutant GISTs expressed distinct immune-related profile, suggesting the heterogeneous responses to immune checkpoint blockade may be related to tumor cell-intrinsic biology.

To date, a few trials have been undergone to explore the immune response in GIST. Two published trials reported limited activity of anti-PD-1 blockade [37, 79]. The 6-month non-progression rate is 11.1% among 10 patients with advanced GIST receiving pembrolizumab [37]. Macrophage infiltration and IDO1 pathway infiltration may influence the immune response. A randomized phase II trial of nivolumab alone (N) or with ipilimumab (N+I) in patients with advanced GIST refractory to at least imatinib (median prior lines of 3) reported the 4-month PFS of 42.1% and 31.3% for the N and N+I groups, respectively. The primary endpoint of response >15% was not met [79]. The role of anti-PD-1 blockade in advanced GIST remained unclear. Future studies with different strategies such as combined with standard TKIs (imatinib, avapritinib, sunitinib, regorafenib, and ripretinib) may provide more insights into the management of advanced GIST.

## Conclusions and future challenges

The discovery of imatinib targeting KIT/PDGFRA and following TKIs revolutionize the management of advanced/metastatic GIST over the past two decades. Recent approval of ripretinib provided a treatment option beyond the progression of standard three-line TKI treatment in advanced/metastatic GIST. Avapritinib approved for first-line GIST with PDGFRA D842V resolved the clinical issue of primary resistance to imatinib in this mutation type. The success of the two novel TKIs also bring some insights in the management of advanced GIST. First, avapritinib approved for certain mutation type of PDGFRA reflects the importance of identification of the molecular profile in clinical practice. The molecular profile is able to not only guide treatment options but also provide prognostic information, facilitating the personized therapies for certain ultrarare subset of GIST. Second, the treatment approach after the progression beyond ripretinib remains unmet medical need. Of note, sunitinib, regorafenib, and the fourth-line ripretinib showed limited response (<10%) with the majority of stable disease, indicating the lack of apoptosis ability among all following TKIs after imatinib resistance. In addition to the development of TKI, the treatment strategies may need more novel therapeutic targets to enhance synergistic cytotoxic effects. The emerging TKI, antibody-drug conjugates, and immunotherapy are undergoing clinical trials, which are also important for wild-type GIST. Future investigational therapies and calling for molecular testing may further improve the disease control and quality of life. Finally, management of GIST requires a multidisciplinary team approach, in which surgical resection or other localized therapies combined with systemic treatments is mandatory in early-stage but also advanced diseases.
